# Analysis of Strain and Defects in Tellurium-WSe_2_ Moiré Heterostructures Using Scanning Nanodiffraction

**DOI:** 10.1021/acsnano.3c04283

**Published:** 2023-11-13

**Authors:** Bengisu Sari, Steven E. Zeltmann, Chunsong Zhao, Philipp M. Pelz, Ali Javey, Andrew M. Minor, Colin Ophus, Mary C. Scott

**Affiliations:** † Department of Materials Science and Engineering, University of California Berkeley, Berkeley, California 94720, United States; ‡ The National Center for Electron Microscopy, Molecular Foundry, Berkeley, California 94720, United States; § Materials Science Division, Lawrence Berkeley National Laboratory, Berkeley, California 94720-8099, United States; ∥ Department of Electrical Engineering and Computer Sciences, University of California Berkeley, Berkeley, California 94720, United States; ¶ Institute of Micro- and Nanostructure Research, Center for Nanoanalysis and Electron Microscopy, Interdisciplinary Center for Nanostructured Films, Friedrich-Alexander-Universitat Erlangen-Nurnberg, Erlangen 91058, Germany

**Keywords:** tellurium, WSe_2_, moiré superlattices, scanning nanodiffraction, strain, defects

## Abstract

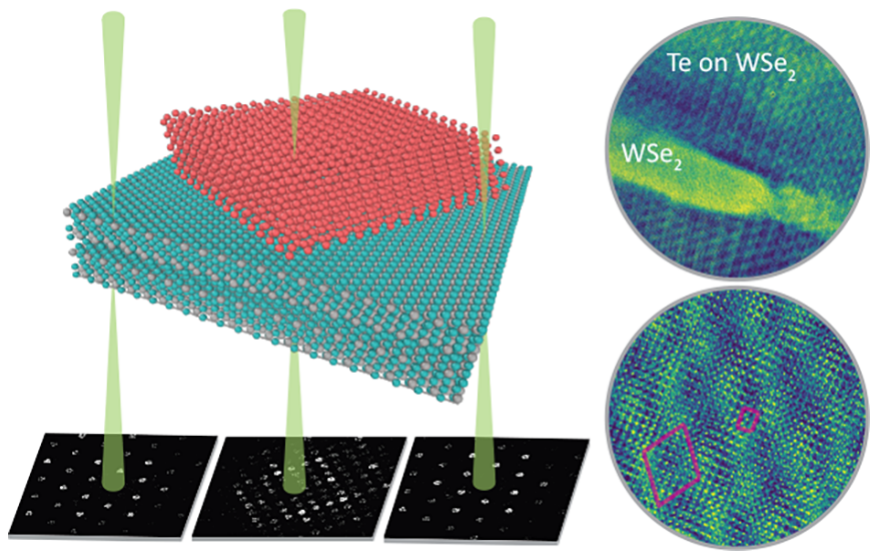

In recent years, there has been an increasing focus on 2D nongraphene materials that range from insulators to semiconductors to metals. As a single-elemental van der Waals semiconductor, tellurium (Te) has captivating anisotropic physical properties. Recent work demonstrated growth of ultrathin Te on WSe_2_ with the atomic chains of Te aligned with the armchair directions of the substrate using physical vapor deposition (PVD). In this system, a moiré superlattice is formed where micrometer-scale Te flakes sit on top of the continuous WSe_2_ film. Here, we determined the precise orientation of the Te flakes with respect to the substrate and detailed structure of the resulting moiré lattice by combining electron microscopy with image simulations. We directly visualized the moiré lattice using center of mass-differential phase contrast (CoM-DPC). We also investigated the local strain within the Te/WSe_2_ layered materials using scanning nanodiffraction techniques. There is a significant tensile strain at the edges of flakes along the direction perpendicular to the Te chain direction, which is an indication of the preferred orientation for the growth of Te on WSe_2_. In addition, we observed local strain relaxation regions within the Te film, specifically attributed to misfit dislocations, which we characterize as having a screw-like nature. The detailed structural analysis gives insight into the growth mechanisms and strain relaxation in this moiré heterostructure.

Two-dimensional (2D) materials research has undergone explosive growth in the past decade, with applications in flexible electronics and optoelectronics,^1^ catalysis,^2^ biomedicine,^3^ and environmental science.^4^ More recently, further breakthroughs came from stacking and twisting two or more layers of 2D materials. This creates what are known as moiré lattices, which exhibit periodic length scales larger than that of the atomic lattice spacings.^5^ Thanks to the introduction of this periodicity, these materials show considerable deviations from their expected electronic, optical, and magnetic phenomena, such as flat bands, moiré excitons, surface plasmon polaritons, interlayer magnetism, superconductivity, and 2D ferroelectricity.^6,7^ Various two-dimensional (2D) materials have been studied by stacking the same, or different, 2D crystals to produce functional devices.^1,6^ 2D materials such as graphene, hexaboron nitride, and transition metal dichalcogenides (TMDCs) can act as building blocks for these heterostructures, creating a nearly infinite design space for moiré heterostructures.

Although the ability to tune a heterostructure’s optoelectronic performance as two or more layers are stacked is desirable, the strain transfer between different van der Waals layers is expected to induce dramatic changes in a material’s electronic, quantum transport, and photonic performance as the complexity of the structures increases.^8−10^ In order to scale up the applications of such heterostructures, there is a great need to understand how strain develops and relaxes during thin film growth and how it behaves after the growth. Although the primary strain relaxation mechanism in heterojunctions is the formation of misfit dislocations, there are additional strain relaxation pathways for the 2D films. Recent experimental works suggest that misfit dislocations alone cannot account for the measured strain relaxations in heterostructures such as tungsten disulfide WS_2_, tungsten diselenide WSe_2_, graphene, and boron nitride (BN). Instead, out-of-plane ripples play an important role in compensating the local strains.^11,12^ Moreover, de Jong et al. demonstrated that moiré lattices show subtle distortions due to local variations in twist angle and interlayer strain. They also found that the moiré lattice could play a role in stabilizing these defects by minimizing the local stacking fault energy within the moiré unit cell.^13^ Therefore, mapping local strain and defect concentration is an important step toward fully understanding the behavior of heterostructure materials.

Recently, Te thin films have been used in electronics, optoelectronics, energy devices, and sensors due to their inherent structural anisotropy, high hole mobility, and large photoconductivity.^14−16^ Distinct from 2D van der Waals materials such as TMDCs, crystalline Te is composed of an array of covalently bonded parallel atomic chains on a two-dimensional (2D) hexagonal lattice parallel to the [0001] direction. The bonding between the nearest-neighbor atoms in the chain is weak van der Waals (vdW) bonding. The appealing properties of tellurium such as anisotropic carrier mobility,^17^ thermal conductivity,^18^ and mechanical and electromechanical properties^19^ have fueled a resurgence of interest in synthesizing ultrathin Te films. Huang et al. demonstrated that, when monolayer tellurium is placed on a graphene substrate (with the tellurium chains aligned parallel to the graphene surface), it exhibits a significant band gap of around 1 eV. As the thickness of tellurium increases, the band gap gradually decreases and approaches the bulk value of 0.34 eV.^20^ This axis- and thickness-dependent behavior reveals unexplored opportunities for tuning the optical and electronic properties of tellurium-based materials for real devices and thin film applications. G. Hao et al. stated that ultrafast solid-state lasers, along with all-optical modulation and various other nonlinear devices, can be developed by leveraging Te-transition metal dichalcogenide (TMDC) heterostructures.^21^ Research and development in low-dimensional tellurium production is ongoing to explore and understand the full potential of these materials for various technological applications. As the demand for advanced electronic and optoelectronic devices, energy storage solutions, and efficient catalysts increases, the significance of low-dimensional tellurium materials will likely continue to grow. However, the in-plane orientation of the Te crystal on most substrates is random. Previously, we demonstrated van der Waals epitaxial growth of 2D Te flakes with thickness down to 5 nm on the surface of 2D transition metal dichalcogenides (TMDCs) WSe_2_, WS_2_, MoSe_2_, and MoS_2_ flakes. In the case of WSe_2_, the *c*-axis of Te is aligned with the armchair direction of the substrates.^22^ The ability to control the growth direction of the Te thin films is exciting; however, it is still necessary to understand the strain and strain relaxation mechanisms which may alter the behavior of this system.

The formation of a moiré structure can lead to two distinct scenarios: one wherein the two layers share a mutual (expanded) periodicity and the other wherein they do not. Moiré structures with a mutual periodicity are termed “commensurate”, and aperiodic structures are designated “incommensurate”. Incommensurate and commensurate structures can coexist within the same system simultaneously. In addition to these moiré structures, approximate periodicities may be present, e.g.. rational approximates of an irrational periodicity may be observable. These periodicities are also experimentally observable, and we term them “approximate tilings”. Various characterization techniques are employed to reveal moiré lattices with varying length scales, including convergent beam electron diffraction (CBED),^23^ scanning tunneling microscopy (STM),^24^ atomic force microscopy (AFM),^25^ and differential phase contrast (DPC) and four dimensional-scanning transmission electron microscopy (4D-STEM).^26^ Out of these methods, 4D-STEM, which records a 2D image of the diffracted electron beam at each probe position, gives valuable insights into the moiré lattice structure, orientation, and structure-dependent properties.^27,28^ 4D-STEM can also be used to map the strain fields, allowing a large field of view and flexibility with regard to sample type and orientation.

In this study, we performed a detailed analysis of the WSe_2_–Te system to study the structure and orientation of the moiré lattice formed due to the interaction between the substrate and the film. We determined the precise orientation of the Te flakes with respect to the substrate by combining electron microscopy with image simulations, showed that Te chains are aligned along the armchair direction of WSe_2_, and deduced how the moiré superlattice is formed. We directly imaged the incommensurate moiré lattice and found an approximate periodic tiling that forms a periodic moiré cell with CoM-DPC and STEM imaging. Mapping strain and detecting defects in the films is nontrivial; for this, we used scanning electron nanodiffraction with subsequent strain and defect contrast  analysis. Our results indicate nonuniform strain in both the Te and WSe_2_ films, significant rotation in portions of the Te film, and dislocations associated with the rotated regions of the Te film.

## Results/Discussion

**Figure 1:**
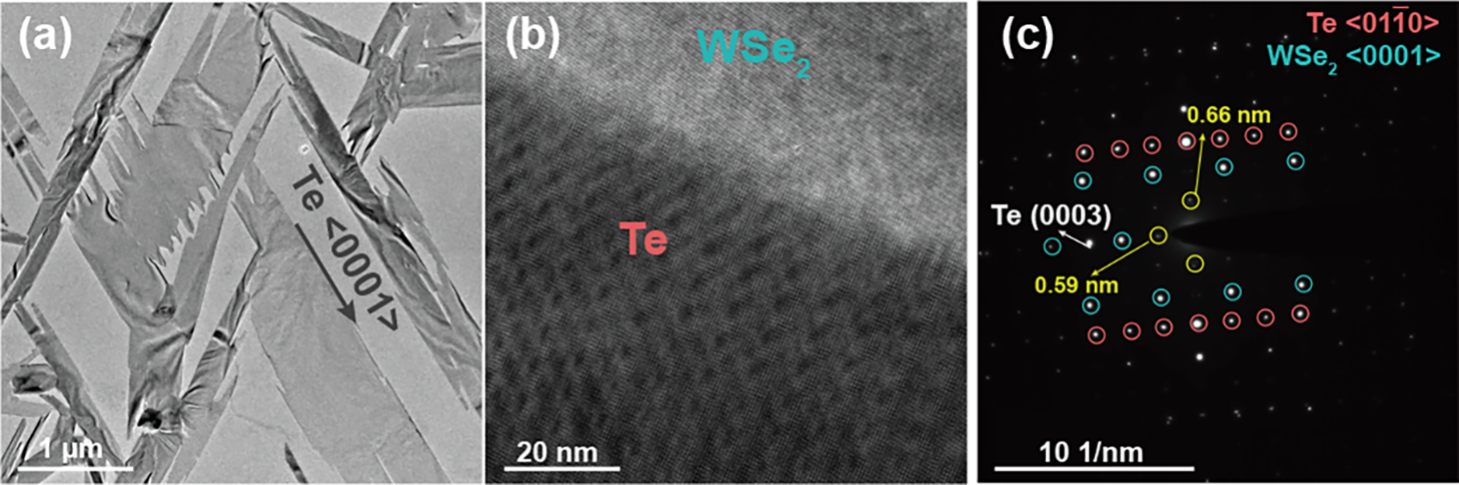
(a) Bright field image of the Te flakes on WSe_2_ substrate. (b) HRTEM images showing the moiré lattice. (c) SAED pattern collected from the HRTEM region shown in (b).The yellow circles indicate the additional modulations that result from the approximate tiling.

**Figure 2:**
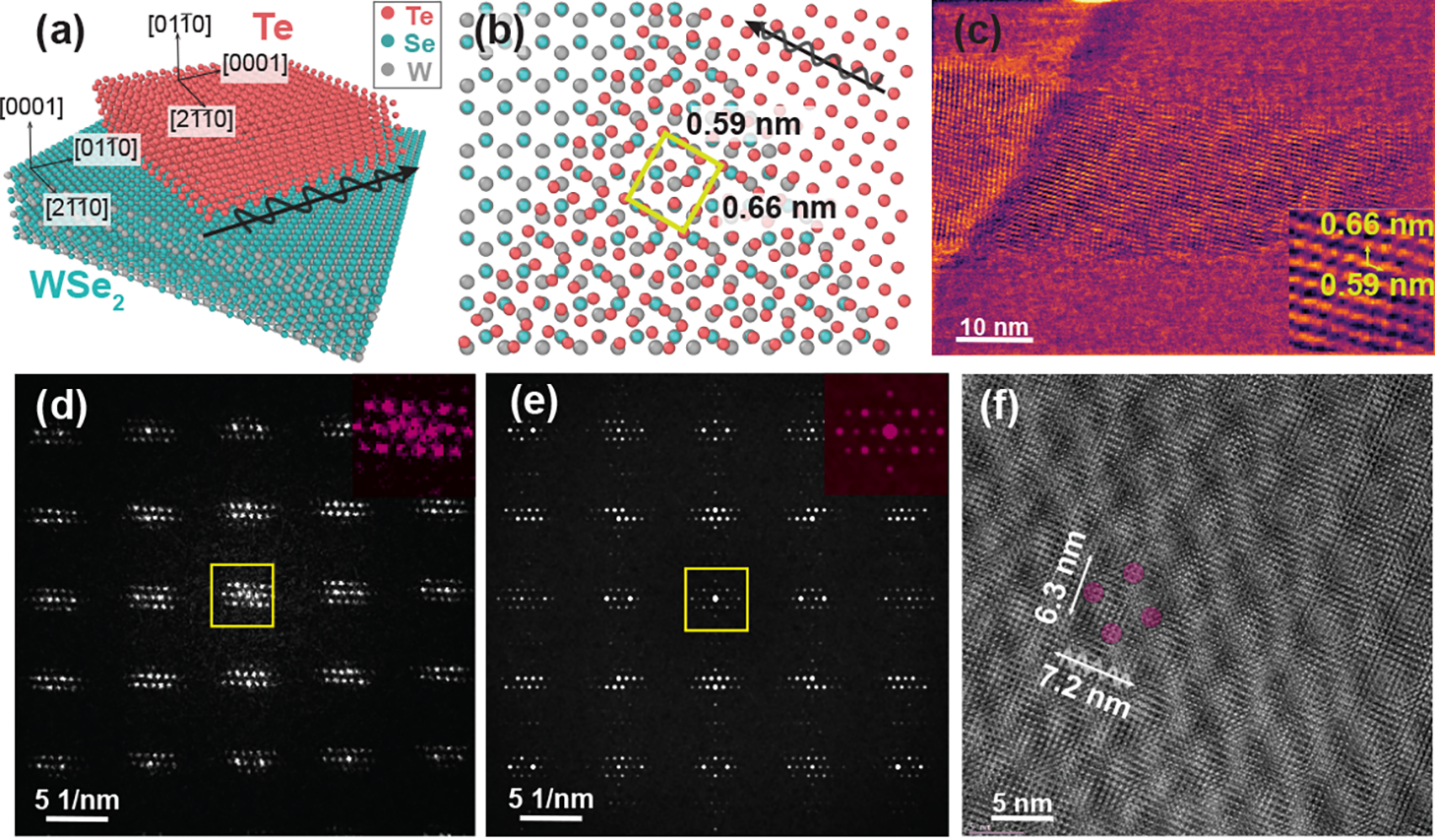
(a) Schematic of the aligned Te deposited on WSe_2_. Pink atoms represent Te, green represent Se, and gray are W. (b) Atomic configuration between Te atomic chains and WSe_2_ surface where the *c*-axis of Te is parallel to the armchair direction of WSe_2_. The yellow square shows the approximate tiling. (c) CoM-DPC image indicating the periodic patterns of the approximate periodic tiling. (d) FFT of the HRTEM image in (f). The inset shows the magnified image of the center part of the FFT. (e) FFT of the simulated HRTEM image. (f) HRTEM image showing the moiré lattice with two basis vectors with lengths of 7.2 and 6.3 nm.

The overview TEM images and diffraction data shown in Figure 1 confirm the preferential growth direction of the tellurium flakes on the WSe_2_ substrate. They typically have one smooth facet termination and jagged edges along the <0001> direction, as shown in Figure 1(a). The thickness of the flakes is about 12 ± 2 nm. The Bragg peaks corresponding to each material in the SAED pattern are shown in Figure 1(c), with Te Bragg peaks labeled pink and WSe_2_ in blue. The HRTEM data in Figure 1(b) and the SAED data in Figure 1(c) indicate that the Te and WSe_2_ films are aligned with the <011̅0> and <0001> zone axes along the beam direction Figure 2(a). The relative orientation of the Te and WSe_2_ films is consistent with our previous observation that the Te chains are aligned with the armchair direction of the WSe_2_, due to the relative binding energies of the materials.^22^ We show the orientation of WSe_2_ and Te in Figure 2(b), where the two atomic structures overlaid in the middle part of the simulated supercell structure.

In addition to the main Bragg peaks of both phases, there are extra intensity modulations generated by the interaction between the WSe_2_ and Te films (Figure 1(c)) shown as yellow circles. We also observed satellite peaks associated with the Bragg peaks indicated by the yellow circles in Figure 1(c). These peaks are highlighted within the yellow squares in Figure S1. The peaks with the larger periodicity correspond to an incommensurate moiré lattice formed between the Te and WSe_2_ films, which is represented by purple arrows in Figure S1(b). Similarly, the peaks indicating a smaller periodicity, highlighted by yellow circles in Figure 1(c) and corresponding to the main Bragg peaks visible in Figure S1(b), are attributed to approximate periodic tiling.

The relative dimensions of the observed incommensurate moire lattice and the smaller approximate tiling are shown in Figure S2. The smaller, visibly periodic pattern, or approximately periodic tiling, which constitutes the dominant visual feature in experimental observations (as shown in Figure 1(b) and (c)), emerges due to the minimal separation between the true tiling and its approximate counterpart. This phenomenon is illustrated by the yellow circles in Figure S2(a) and (b), a consequence of illustrative rendering, and in our diffraction and other data sets, owing to the physical dimensions of atomic potentials. The moiré periodicity associated with the specific angle of this system is comparatively large, necessitating a broader field of view for visibility, as depicted by the purple circles in Figure S2(c). To elucidate the expansion of the incommensurate moiré lattice, we further illustrate the larger periodicity inherent in the approximate tiling, as shown in Figure S2(d).

To directly visualize the outcome of the tiling in real space with atomic resolution, we utilized a combination of HRTEM imaging, HRTEM simulation, and DPC/STEM. Atomic resolution DPC imaging relies on the deflection of the focused beam by the electrostatic potential of the atoms in the sample.^27^Figure 2(c) shows CoM-DPC images that indicate the approximate periodic tiling, consistent with the *d*-spacing of the extra peaks (yellow circles) observed in the SAED in Figure 1(c). We also showed the same periodicity in Figure 2(b) with a solid square.

In addition to the extra intensity modulations formed due to the approximate periodic tiling, we detected an incommensurate moiré superlattice in the HRTEM images (Figure 1(b) and Figure 2(f)), and it is faintly visible in the CoM-DPC/STEM image (Figure 2(c)). We confirmed the observation of this periodicity by the satellite peaks appearing in the fast Fourier transform shown in Figure 2(d) and in the inset in Figure S1(b).

To unravel the structure and orientation of the two films that form the moiré lattice, we performed a two-stage image simulation. Electron microscopy observations established the relative in-plane orientation of the materials. The specific orientation of tellurium was determined through controlled rotation along the *c*-axis to facilitate the determination of a smaller periodicity via image simulations. Then, an iterative fitting simulation incorporated experimental data and prior simulations to solve for the most accurate relative positions of the two materials and thereby determined the moiré structure. Through iterative image simulations, we adjusted parameters such as rotation, strain, shear, and thicknesses of the Te and WSe_2_ films. The simulated fast Fourier transform (FFT) images remained unaffected by aberrations and material constraints (Figure 2(e)). We report how Te and WSe_2_ unit cells must be stacked in terms of rotation and lengths in Table 1. According to iterative simulation results, the best approximate match we found to form the moiré lattices at the larger scale, which is shown in the HRTEM image in Figure 2(f), is to repeat the Te unit cell 19 times along the <21̅1̅0> axis, 12 times along the <011̅0> axis and WSe_2_ unit cell, 14 times along the <21̅1̅0> axis, and 12 times along the <0001> axis. Iterative fitting yielded a rotation of 0.14 degrees and minor strain in satellite peak rows within the experimental FFTs to construct the incommensurate moiré lattice from the cell parameters. Notably, moiré satellite peak positions exhibited complex dependence on the parent lattice. Each peak “cluster” displayed slight rotational shifts, with significant internal cluster rotation. Figure S3 visually presents the simulated FFT and reciprocal lattices (Figure S3(c) and (d)) resulting from iterative simulations, originating from the experimental FFT in Figure S3(a).

**Table 1:** Crystallographic Directions Corresponding to the Axes of the Orthogonal Supercell Used for Simulations with the Corresponding Cell Dimensions^a^

Material	*a*	*b*	*c*
WSe_2_	(21̅1̅0)	(011̅0)	(0001)
	3.3270	5.7625	12.9600
Te	(21̅1̅0)	(0001)	(011̅0)
	4.5152	6.0026	7.8064

a The electron beam propagates along the *c* direction of the cell. All dimensions are in Å.

**Figure 3:**
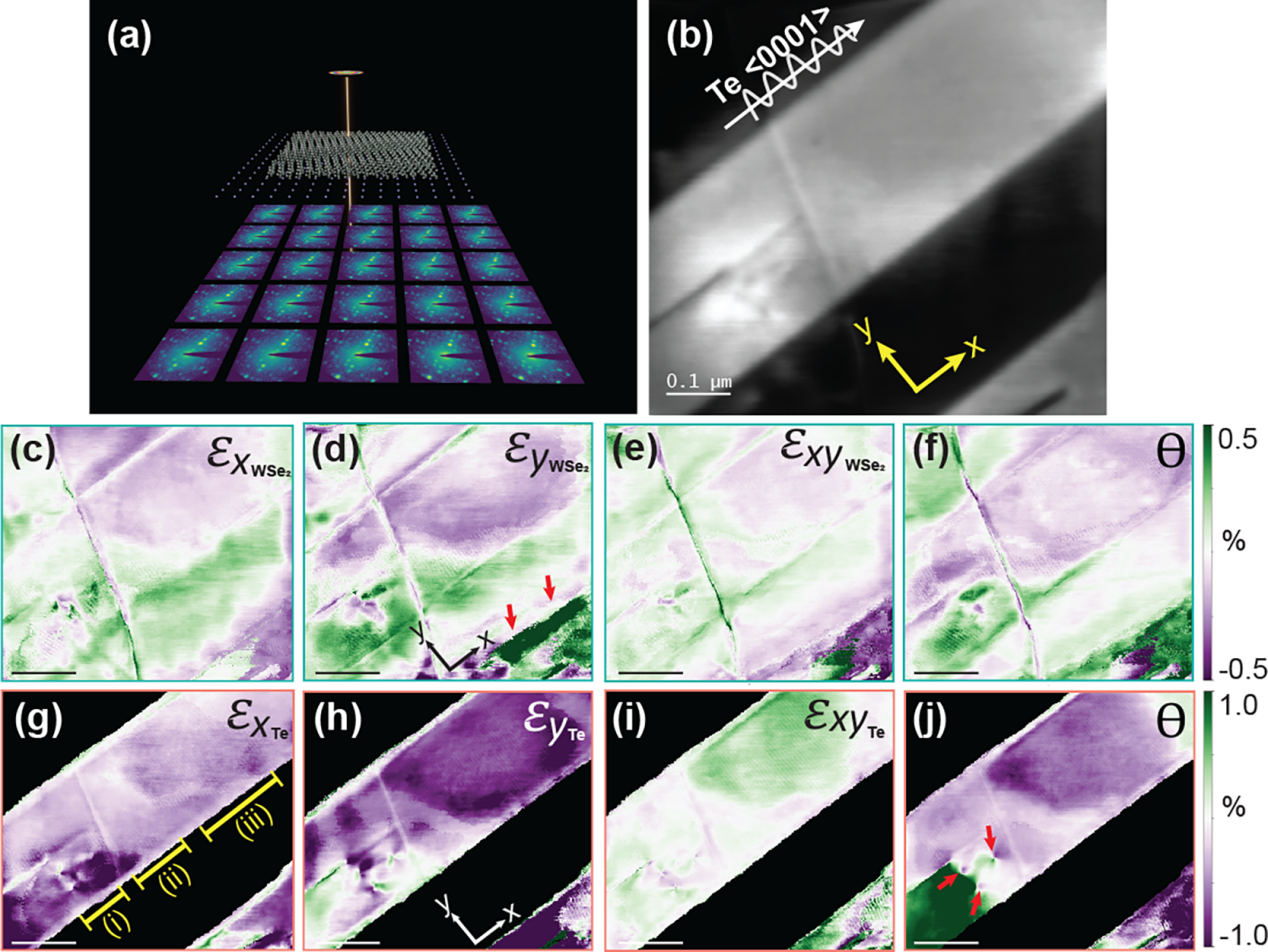
(a) Sketch of experimental setup for 4D-STEM strain mapping. A converged electron probe is rastered across the sample, and a diffraction pattern is collected for each position. (b) HAADF image of the flake and the substrate. Strain maps generated from (c–f) WSe_2_ and (g–j) Te lattices. We defined the reference lattice as the median measured lattice constants of WSe_2_ and Te and used these values to calculate the relative strain values. The *x* and *y* components of the strain, for which the direction is illustrated in (b), (d), and (h), correspond to the <0001> and <12̅10> directions of the Te lattice and the <21̅1̅0> and <011̅0> directions of the WSe_2_ lattice. Scale bar is 0.1 μm.

4D-STEM can detect local changes in the lattice at each scan position by first measuring a 2D diffraction pattern at each probe location (Figure 3(a)). Local strains are then mapped throughout the sample by measuring the infinitesimal change in the lattice with respect to the reference lattice. The results of 4D-STEM strain mapping are shown in Figure 3. We generated separate strain maps for WSe_2_ and Te by first individually detecting their Bragg peak positions, as shown in Figure S4(e) and (g), and then calculating the local strain for each material from their nonoverlapping Bragg peaks to get individual strain maps Figure 3). The maps of the *x*, *y*, and shear components of the strain tensor, as well as the principle rotation, are shown in Figure 3(c–j). Since we are aiming to understand the strain relaxation along the Te chain and the direction perpendicular to the Te chains, we calculated the strain components along these two lattice vectors, labeled *x* and *y*, respectively. Therefore, the ε_*x*_ component of strain in each material can be understood as strain along the *c* axis of Te, and the ε_*y*_ component is the one perpendicular to the *c* axis of the Te.

As shown in Figure 3(c–j), the strain solely in the WSe_2_ layers is lower (±0.5%) than on the Te film (±1%). The strain map shown in Figure 3(d) indicates an in-plane tensile strain along the *y* direction at the edges of the Te flakes, as shown by the red arrows. This indicates that the van der Waals forces of Te along the direction perpendicular to the chain cannot compensate for the strain generated by the epitaxial growth of Te films. The reason behind this stems from the surface energy difference between different planes of Te. The bond strength along the *c*-axis of Te, and hence the surface free energy of the {0001} surfaces, is between two and three times greater than the corresponding values for the prismatic {1010} surface.^29^ In order to decrease the surface energy, the surface areas of the Te {0001} facets are limited. Te crystals, therefore, tend to grow in the form of extended hexagonal prisms or dendrites parallel to the substrate surface. We also observed the same behavior in other Te flakes too, as shown in Figure S5. Another reason for the dendritic growth can be explained from the kinetics point of view: since the Te interchain bonds are primarily of van der Waals character, the edge diffusion will be very rapid relative to the diffusion of Te atoms on a pristine {0001} surface on which the atoms are covalently bonded to the chains.^22^ Therefore, tellurium tends to grow as flakes and have rough {0001} facets.^30^

Because of the difference in the *d*-spacings of the substrate and film along the chain (*d*_*WSe*_2__ = 0.571, *d*_*Te*_ = 0.596),^11,31^ compressive strain is expected to be dominant along the *x* direction (the Te chain direction) in the strain maps. To better understand the strain relaxation mechanisms in the heterostructure, we divided the strain maps into three regions, marked as (i), (ii), and (iii) based on the observed strain and rotations (marked on Figure 3). At regions (ii) and (iii) of the strain map labeled with yellow lines, the Te flake releases the strain through lattice distortions. The periodic lattice pattern that is also visible on both HAADF and the right-end of the strain maps matches the moiré lattice we observed in Figure 2(f) and Figure S4(b) and (c).

The leftmost region, region (i), indicated in Figure 3(g) shows both a compressive strain of approximately 0.5% and significant rotation (Figure 3(j)). The linear feature that separates the left (i) and middle parts (ii) of the Te flake is visible in the HAADF image (Figure 3) and strain maps obtained from the WSe_2_ lattice vectors (Figure 3). It is likely either a dislocation or a crack on the substrate. Although we do expect that the substrate releases the strain during the preannealing treatment, the substrate may be exposed to stress during the transfer of the substrate and the film to the TEM specimen holder. Furthermore, in our efforts to ensure there was no obvious mechanical damage resulting from the TEM sample preparation, we conducted thorough examinations at both lower and higher magnifications in bright field mode. The strain values we measured for WSe_2_ substrate and Te are between −0.5 to 0.5% and −1 to 1%, respectively, and we attribute these modest strain values to Te–WSe_2_ interactions and expect much higher strain values for mechanically damaged samples. For example, the 5 layers of WSe_2_ can endure 12.4 GPa stress and 7.3% strain without fracture or mechanical degradation.^32^ There is an approximately strain-free region at the midpart region (ii) close to the linear defect, which suggests there is another strain relaxation mechanism than the lattice strain that helps the lattice overcome the stress generated due to the linear feature and rotation of the left part of the flake. After careful investigation of region (i), which contains significant rotation of the Te lattice, we detected misfit dislocations, which can be shown as red arrows on the rotation maps. The misfit dislocations are more easily recognized in the rotation map by observing their dipole fields as in Figure 3.^11^ The dislocations in crystals composing the heterostructure, in the form of moiré pattern dislocations, stem from a missing row of atomic unit cells. The appearance of these dislocations is magnified with the addition of layers. Therefore, moiré pattern dislocations contain features similar to those of dislocations in Te crystals.

**Figure 4:**
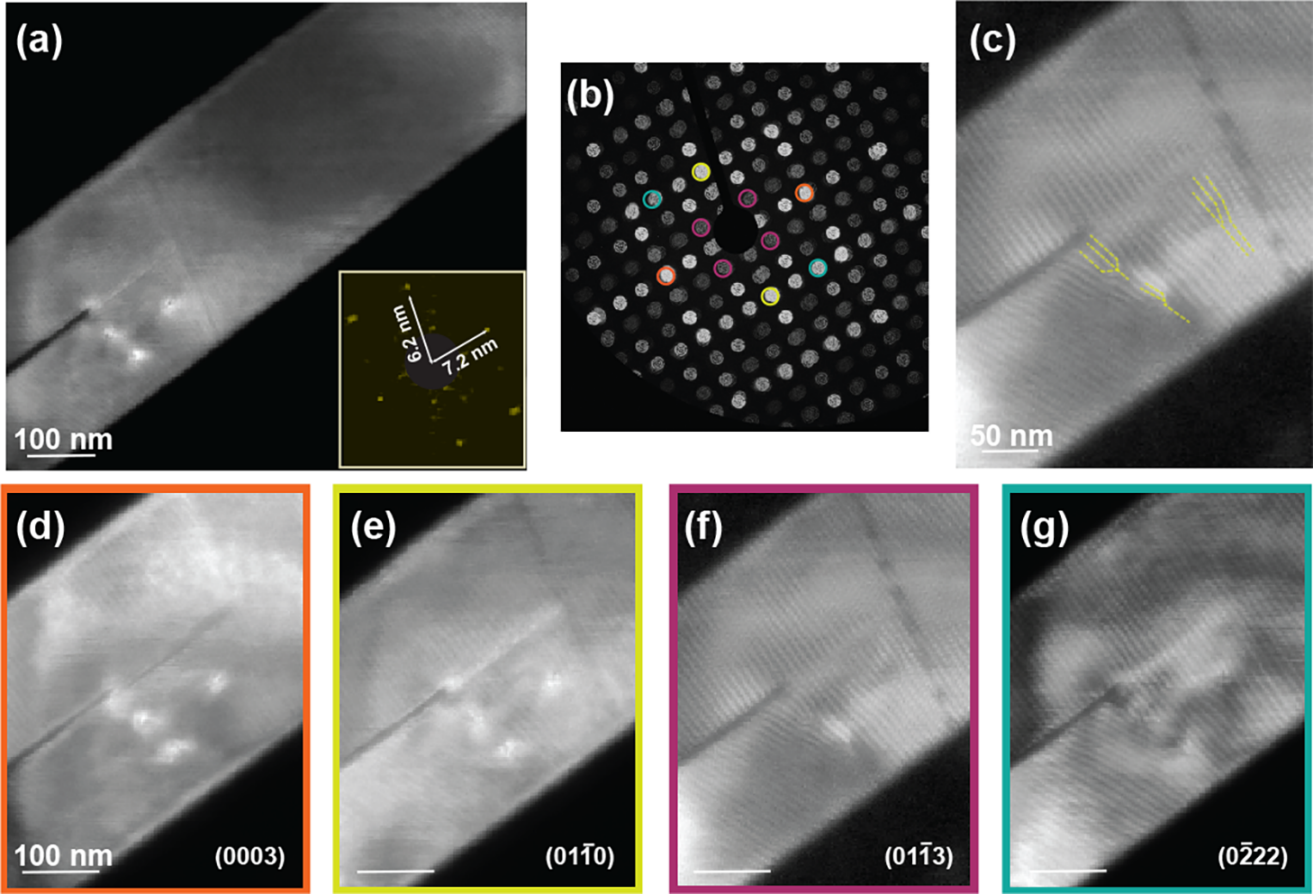
(a)Virtual dark field image. (b) Virtual annular dark-field detectors. (c)Virtual dark field image showing moiré dislocations. (d–g) Virtual dark-field images corresponding to circular detectors about each of the indexed Bragg peaks.

To measure the Burgers vector b⃗ of the dislocations, we performed virtual dark-field image analysis and subsequent defect contrast analysis.^33^ In Figure 4, we demonstrate that the dislocations are visible on planes along the *c*-axis and perpendicular to the *c*-axis. However, their contrast disappears for the planes corresponding to the moiré lattice. We found that the (02̅22) set of planes of the Te lattice are where the dislocations are least visible. From the invisibility criterion of , the Burgers vector is parallel to the <1̅21̅3> direction. This reveals that the Burgers vector is one of the most common ones in Te, b⃗ = *c* + *a*.^34^ Dislocations which are located in the prismatic planes and lead to a rotation around the <21̅1̅0> axis, as in our system, are connected with two different dislocation families which are “*c*” screws and one of the three “*a*” screws.^35^ However, previous work argues that the invisibility criterion of  is not a sufficient condition for the identification of the dislocations in Te due to elastic anisotropy.^34^ They added that the condition is sufficient only when the displacement field of the dislocation is parallel to the dislocation line, as in the case of screw dislocations. For this reason, the moiré dislocations we observed in Figure 4 likely have a screw character, and the Burgers vector is not precisely parallel to the beam direction, <21̅1̅0>.

The observed topological dislocations break the translational symmetry of the moiré lattice and are expected to alter the properties of the system; for example, they may cause a phase difference between the electron paths encircling the defect clockwise and counterclockwise.^13^ In addition, the observed strains in WSe_2_ and Te are known to alter the optoelectronic properties. In the case of WSe_2_, biaxial strain bends down both the conduction band minimum and valence band maximum at different rates, leading to an overall bandgap narrowing.^36^ In trigonal Te, shear (hydrostatic or uniaxial) strain causes the material to change from a trivial insulator to a strong topological insulator.^37^ Ultimately, both the observed defects and strain will influence the behavior of this moiré heterostructure.

## Conclusions

Moiré heterostructures are intensely studied due to the attractive possibility of tuning their optoelectronic properties by varying the lattice mismatch and orientation. However, defects and strain may further alter the material performance, and it is therefore important to monitor these structural features that may arise during film growth. Here, we examined Te-WSe_2_ heterostructures using electron microscopy. PVD growth of Te on WSe_2_ produced thin Te films with chains oriented along the armchair direction of the WSe_2_. We determined the moiré structure of this system by solving for the orientation of the Te and used scanning nanodiffraction to measure strain in both materials and to detect defects in the Te. The strain and defect formation arise from the lattice mismatch between the two materials, and geometrically necessary defects occur in Te as a part of the film growth process. Ultimately, our findings not only elucidate how moiré structures form due to the interaction between Te and WSe_2_ materials but also reveal exciting prospects for studies on moiré and/or strain engineered layered materials that provide a platform for engineering and manipulating materials to develop functional materials and devices both at macro- and nanoscale by using WSe_2_ and Te materials. This study illustrates how prevalent local structural imperfections can be in van der Waals heterostructures produced by thin film deposition and points to the possibility of further tuning growth conditions to produce more perfect films. Ultimately, local measurements of defects and strain are important tools to provide insight into the production and performance of van der Waals heterostructures.

## Methods/Experimental

### Materials Growth and TEM Specimen Preparation

We exfoliated the WSe_2_ flakes on SiO_2_/Si substrates. For the growth of the thin film, we used a two-zone hot wall quartz tube where we loaded the alumina boat containing powdered Te (99.999%, Sigma-Aldrich) as the precursor in one zone and the substrates in the downstream region. We preannealed the substrates at 300 °C under Ar flow to produce more uniform and thicker films. After the pretreatment was performed, we set the substrate temperature up to 130 °C for growing the Te on the WSe_2_ substrate. More details regarding the growth can be found in our previous article.^22^ We transferred the WSe_2_/Te flakes onto a carbon coated TEM grid using a dry transfer method.

### TEM Data Collection and Image Simulations

For the bright-field and high resolution TEM (HRTEM) imaging, we used an FEI Titan 60–300 microscope with an acceleration voltage of 300 kV. We determined the thickness of the films and substrate by using electron energy loss spectroscopy on an FEI Tecnai operated at 200 kV with a C2 aperture of 150 μm, a camera length of 42 mm, and an entrance aperture of 2.5 mm. We used the zero-loss peak to calculate the film thickness within the Digital Micrograph software. The Fourier log deconvolution indicated a *t*/λ value of 0.282 from substrate and 0.4235 from the film which correspond to thicknesses of 18 ± 2 nm and 12 ± 2 nm for the substrate and films, respectively.

To determine the orientation of the Te flakes with respect to the substrate, we matched simulated HRTEM images and their corresponding FFTs to our experimental HRTEM data using custom Python and MATLAB scripts. First, we constructed Te crystals with different zone axes and in-plane orientations on the substrate and performed HRTEM image simulations using the abTEM simulation package.^38^ After we found the in-plane orientation of Te with respect to WSe_2_, we studied the structure and orientation of the moiré lattice. We generated a supercell with Te (*a* = 4.456 Å, *c* = 5.921 Å)^39^ and WSe_2_ (*a* = 3.282 Å, *c* = 12.96 Å)^40^ crystals. To replicate the experimental FFTs, we oriented Te along <21̅1̅0> and tilted WSe_2_ along <0001> zone axes. Then, we rotated the Te crystal along the out-of-plane (*z*) axis to align the chains along one of the armchairs of WSe_2_. After we made the supercells, we simulated HRTEM and the corresponding fast Fourier transform (FFT) images. By iterating the image simulation parameters, which are the rotation, *x* and *y* shifts, shear, and thickness of layers, we found the output cell parameters shown in Table 1 and how many cells were needed to generate the moiré lattice. Our best-fit estimate for the overall thickness is 34 nm, which is composed of 12 layers of WSe_2_ (15.5 nm) and 24 layers of Te (18.7 nm). The thickness values derived from iterative simulations represent the optimal conditions for accurately reconstructing and identifying the moiré lattices according to the experimental FFT. The reconstruction involved adjusting the thickness until the best fit was achieved, yet the resulting intensities exhibit only a weak dependence on the thickness. This is because our reconstruction code primarily focused on accurately determining the position of the Bragg peaks, specifically aimed at solving for the moiré unit cell. The thickness needs only to be sufficiently large to accommodate multiple scattering contributing to the moiré peaks observed in our experimental measurements. Consequently, the simulated cell might tend toward larger thicknesses. However, our rigid model did not account for these effects caused by local strain, which introduces deviations from the ideal moiré condition. As a result, the intensities and, consequently, the layer thicknesses are likely to exhibit significant disparities. It is important to note that the provided thickness is an approximation with the understanding that EELS measurements offer a more reliable determination of the material’s true thickness.

### DPC Imaging

We collected 4D-STEM data for DPC measurements on the double-aberration-corrected TEAM 0.5 microscope with the 4D Camera, developed in-house in collaboration with Gatan, Inc. The 4D Camera is a direct electron detector with 576 pixels × 576 pixels and a frame rate of 87 kHz. We collected 4D-STEM data at 80 kV with a 25 mrad convergence semiangle, a beam current of 52 pA, estimated from 4D camera counts. The real-space pixel size is 0.61 Å, with camera reciprocal space sampling of 173.6 μrad per pixel.^41^

### Scanning Nanodiffraction Data Collection and Analysis for Strain Mapping

We collected scanning nanodiffraction data on the double-aberration-corrected TEAM I microscope operated at 300 keV with a convergence angle of 0.9 mrad, a step size of 2 Å, and a camera length of 130 mm. We used bulls-eye apertures to improve the precision of the detected peak positions^42^ (Figure S4(d)). We utilized the py4DSTEM package for the data calibrations and strain mapping analysis.^27^ Calibrations include correcting shifts of the diffraction pattern, calibrating the rotational offset between the real and diffraction space, and calibrating the pixel sizes. After we performed the calibrations, we detected the Bragg peaks from each of the data points to obtain Bragg vector maps (BVMs) (Figure S4(e) and (g)). Then, we extracted the average reciprocal lattice vectors and indexed them for WSe_2_ and the flakes differently. We defined one of the basis vectors to be aligned along the Te *c*-axis and the other to be perpendicular to the chains. We defined the reference lattice as the median measured lattice constants of WSe_2_ and Te. Then, we computed the infinitesimal strain tensor at each beam position by examining the deviation of their local lattice vectors.

### Virtual Dark-Field Image Analysis

To visualize the dislocations and find the Burgers vector for the dislocations, we performed virtual imaging from the scanning nanodiffraction data set. We placed virtual detectors around the specific Bragg disc positions and averaged the diffraction patterns to generate dark-field images.

## Supporting Information Available

SAED data, approximate tiling in the simulated cell, atomic configurations, fast Fourier transforms (FFTs), high-resolution transmission electron microscopy (HRTEM) images, HAADF images, strain maps, single diffraction patterns of the 4DSTEM data, and Bragg vector maps (BVMs) (PDF)
