# Supplementary material for: Analysis of Strain and Defects in Tellurium-WSe2 Moiré Heterostructures Using Scanning Nanodiffraction
Source: ACS Nano. 2023 Nov 13;17(22):22326–33. doi: 10.1021/acsnano.3c04283 (PMC10690779; doi:10.1021/acsnano.3c04283)
Supplement: Supplementary file 1 — nn3c04283_si_001.pdf [file nn3c04283_si_001.pdf]

Analysis of strain and defects in Tellurium-WSe$_2$ moiré heterostructures using scanning nanodiffraction †

Bengisu Sari,[‡,¶,§] Steven E. Zeltmann,[‡] Chunsong Zhao,[‡,§,‖] Philipp M. Pelz,[⊥] Ali Javey,[§,‖] Andrew M Minor,[‡,¶] Colin Ophus,[¶] and Mary C. Scott[*,‡,¶,§]

‡*Department of Materials Science and Engineering, University of California Berkeley, Berkeley, CA, 94720, United States*

¶*The National Center for Electron Microscopy, Molecular Foundry, Berkeley, CA, 94720, United States*

§*Materials Science Division, Lawrence Berkeley National Laboratory, Berkeley, CA, 94720-8099, United States*

‖*Department of Electrical Engineering and Computer Sciences, University of California Berkeley, Berkeley, CA, 94720, United States*

⊥*Friedrich-Alexander-Universitat Erlangen-Nurnberg, Institute of Micro-and Nanostructure Research, Center for Nanoanalysis and Electron Microscopy, Interdisciplinary Center for Nanostructured Films, Erlangen, 91058, Germany*

E-mail: mary.scott@berkeley.edu

† (Analysis of strain and defects in Tellurium-WSe$_2$ moiré heterostructures using scanning nanodiffraction)

Supporting Information

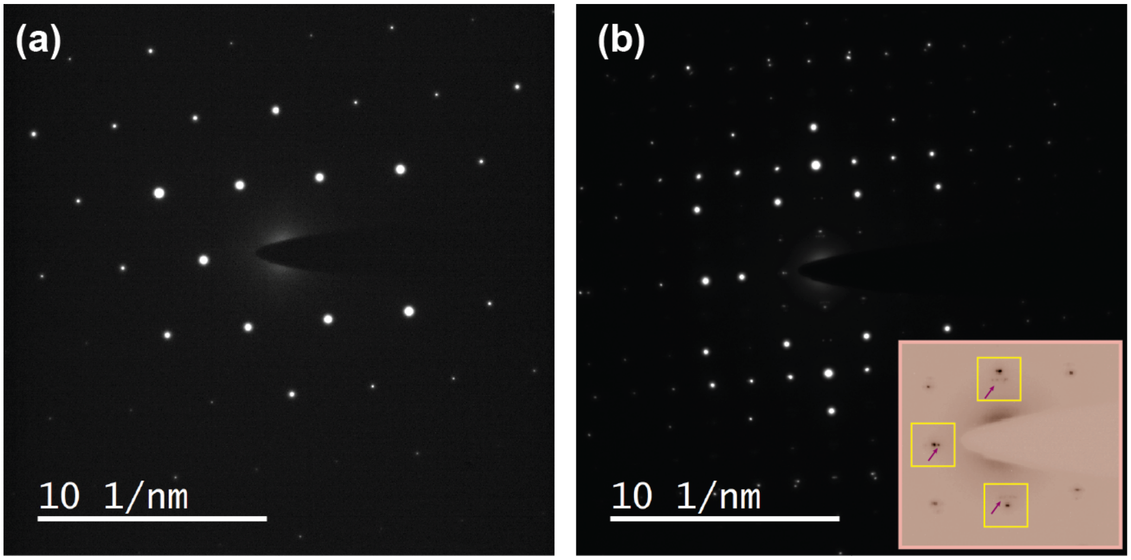

Supporting Information:Figure S1 a)SAED colected from the WSe_2 substrate, b)SAED colected from the flake.The inset displays additional intensity modulations, depicting the approximate tiling as yellow squares, and satellite peaks resulting from the incommensurate moiré lattice, indicated by purple arrows.

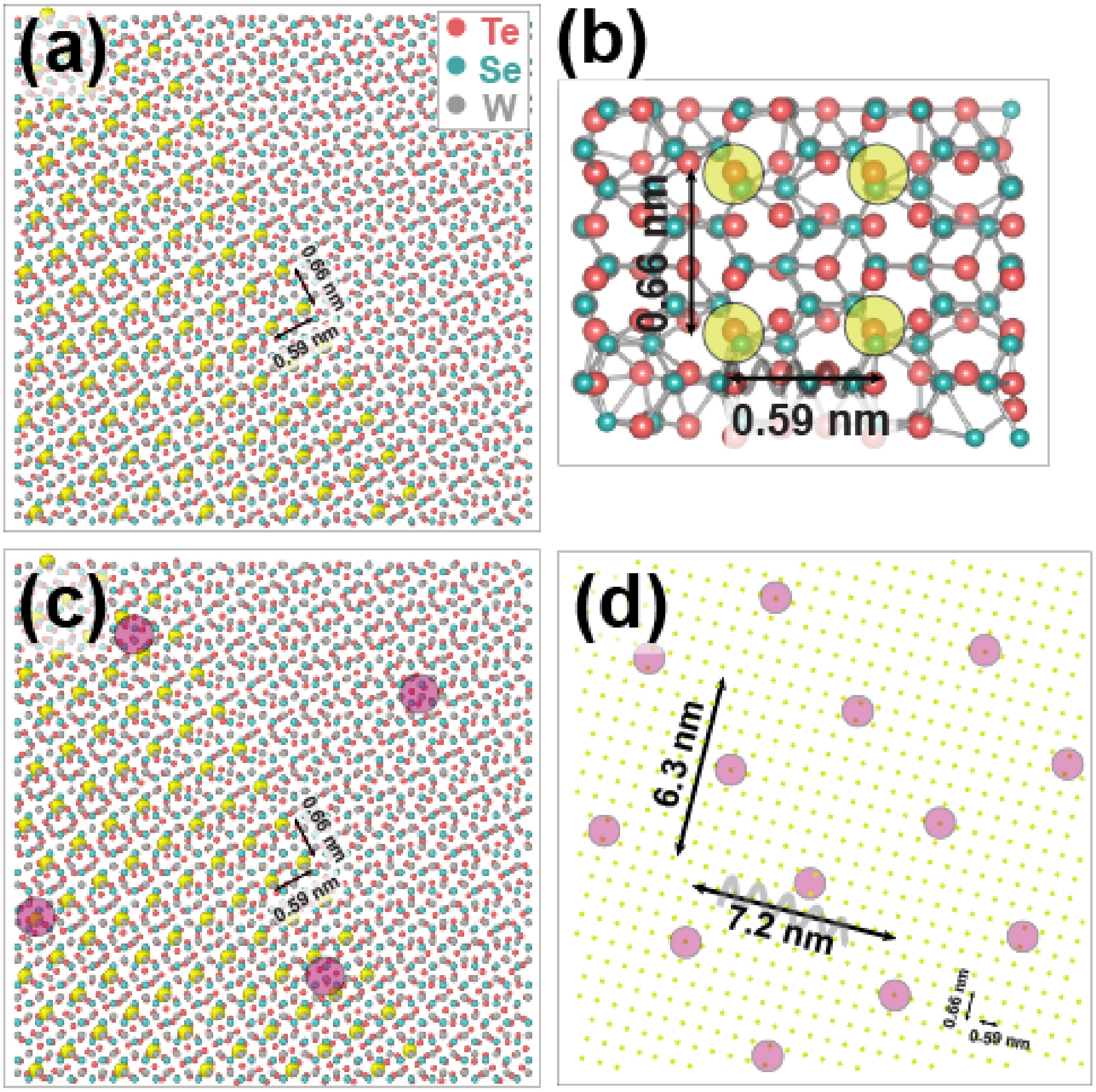

Supporting Information:Figure S2 a) Simulated cell, measuring 80x80 Angstroms, with the c-axis of Te aligned parallel to the armchair direction of WSe2, illustrating the approximate tiling represented in yellow. b) Atomic configuration depicting the overlaid Te and WSe2 lattices, highlighting both the true and approximate tiling. c) Atomic arrangement demonstrating the incommensurate moiré lattice, indicated by purple circles, superimposed on an 80x80 Angstroms simulated cell. d) Atomic configuration of the approximate tiling formed by overlaying the lattices, showcasing the expanded incommensurate moiré lattice. Te atoms are denoted in pink, Se in green, and W in gray.

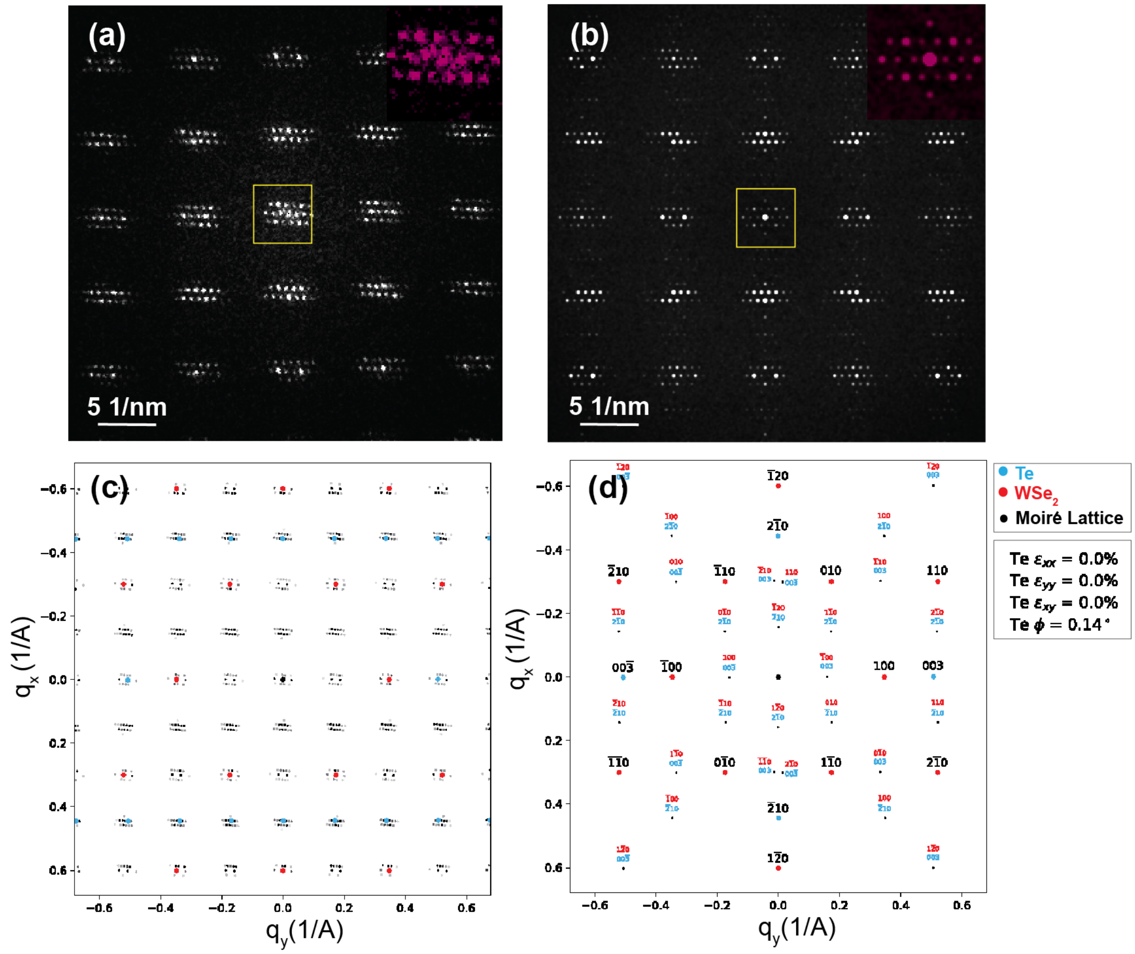

Supporting Information:Figure S3 a) Fast Fourier Transform (FFT) of the high-resolution transmission electron microscopy (HRTEM) image shown in Figure 2f). The inset displays an enlarged view of the central region of the FFT. b) FFT of the simulated HRTEM image. c) Simulated reciprocal lattice demonstrating the impact of a 0.14-degree rotation tilt along the phi or z-axis on each "cluster" of peaks. d) Reciprocal lattice with labeled orientations of WSe2, Te, and moiré lattice peaks, as determined from the simulations

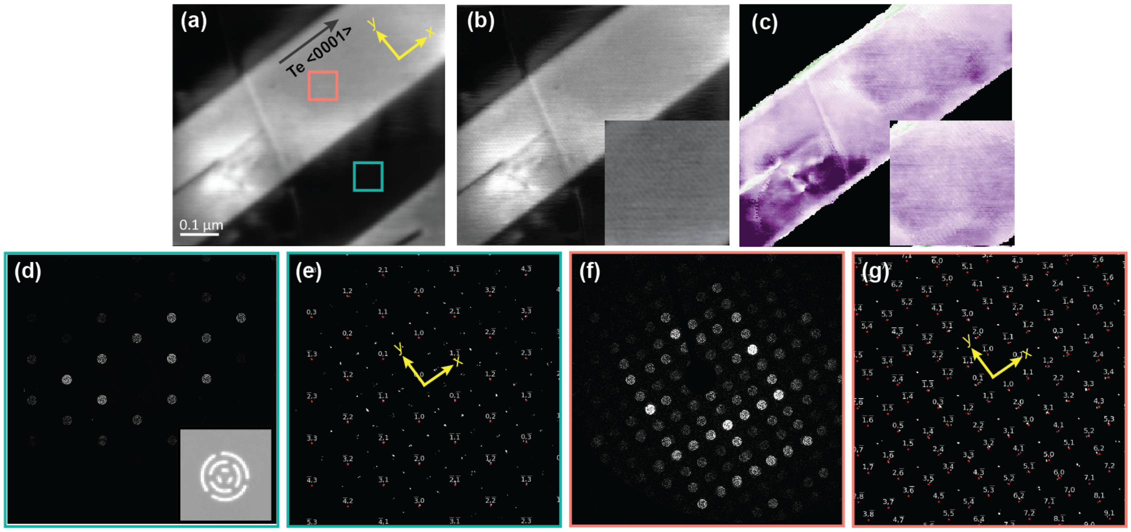

Supporting Information:Figure S4 a)HAADAF image of the flake and the substrate. b) HAADAF image of the flake and the substrate. Inset of the moiré lattice on HAADAF image. c) Strain maps generated from Te lattice vectors. Inset of the moiré lattice on the strain map. d) The mean diffraction pattern of the 4DSTEM data. Bragg vector map (BVM) obtained from the e) WSe_2 and f) Te lattice vectors.

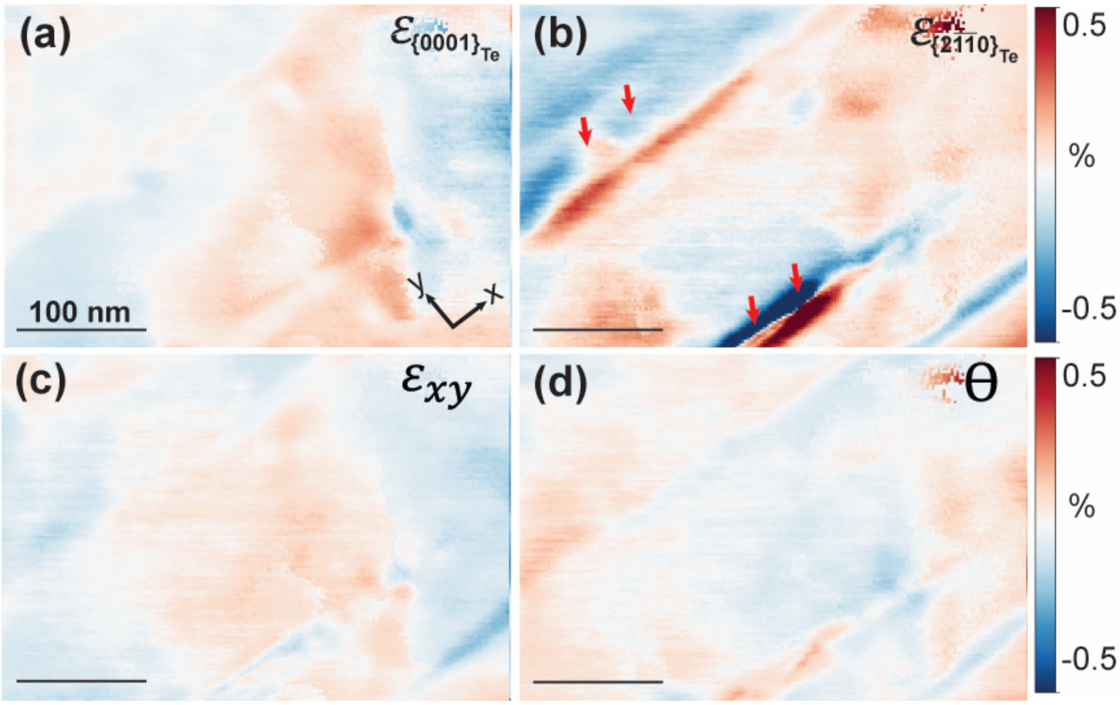

Supporting Information:Figure S5 a-d) Strain maps generated using the WSe_2 lattice from different flakes. b) High tensile strains at the edges of the flakes are indicated by red arrows.
